# Evaluation of the left ventricle contractility during and after 6 months on the ISS using a speckle tracking modality

**DOI:** 10.3389/fphys.2026.1699925

**Published:** 2026-02-11

**Authors:** Philippe Arbeille, Kathryn Zuj, Nicole Vincent, Danielle Greaves, Richard Hughson

**Affiliations:** 1 UMPS-CERCOM. Medical School University of Tours, Tours, France; 2 LIPADE Department of Informatic, University Paris Cité, Paris, France; 3 Faculty of Health, University of Waterloo, Waterloo, ON, Canada; 4 Schlegel-UW Research Institute for Aging, Waterloo, ON, Canada

**Keywords:** contractility, left ventricle, spaceflight, speckle tracking, ultrasound

## Abstract

**Purpose:**

Cardiac contractility can be evaluated during ultrasound evaluations using speckle tracking to map the motion of myocardium during the cardiac cycle. The purpose of the current study was to use this method to evaluate cardiac contractility in astronauts before, during, and after 6 months of spaceflight on the International Space Station.

**Methods:**

Speckle tracking data were recorded from ten astronauts (1F) preflight (PRE), on flight day 150 (FD150) and approximately 4 days after returning to Earth (R4). A 2D apical view video of the left ventricle was captured at each time point for the evaluation of three measures of cardiac contractility: the mean displacement of speckles, the product of mean displacement and the number of speckles tracked, and the size of the area with speckles showing the greatest displacement.

**Results:**

A trend was found for mean speckle displacement to be affected by spaceflight and recovery (p = 0.052) with displacement potentially lower in seven of the ten astronauts on FD150. The product of speckle displacement and number of speckles was significantly lower inflight compared to R4 (p = 0.021). The area with the highest displacement was significantly reduced with spaceflight compared to PRE (p = 0.017). For all variables, values on R4 were not different from PRE.

**Conclusion:**

Results indicate a mild reduction in cardiac contractility with spaceflight. All parameters returned quickly to preflight values by R4, suggesting that these changes are related to a reduction in physical activity and/or fluid blood volume rather than cellular remodeling of the myocardium.

## Introduction

Spaceflight and spaceflight analogue studies have consistently shown physiological adaptations including alterations in cardiac structure and function. Ultrasound evaluations have identified changes in systolic and diastolic volume, stoke volume, and myocardium thickness (Caiani et al., 2014; Dorfman et al., 2007; Perhonen et al., 2001; Negishi et al., 2017). Using speckle tracking, myocardium contractility was also found to be reduced after 21 days of head-down bedrest, an analogue of spaceflight (Greaves et al., 2019). However, cardiac contractility has not been evaluated with long duration spaceflight.

In clinical practice, cardiac contractility is commonly evaluated visually and subjectively on 2D ultrasound video of the heart in a 4-chamber view or indirectly using Doppler tissue imaging (Ziółkowska et al., 2017). Contractility can also be objectively quantified using the speckle tracking method (Caiani et al., 2007; Perez de Isla et al., 2010; Pérez de Isla et al., 2011; Seo et al., 2011; Arbeille et al., 2012 ) and is widely available on many 4D ultrasound systems. Using this method, the ultrasound system generates a 3D time-lapse video of the left ventricle beating, and identifies speckles, which are points of heterogeneity, present in all black and white cardiac ultrasound images. Specific programs have been developed for tracking these points during the cardiac cycle and measuring their respective displacement inside the myocardium. When speckle tracking is activated, the ultrasound system tracks the movement of speckles inside the myocardium during the cardiac cycle (contraction and relaxation) and displays these movements as color overlaid on the left ventricle contour.

Speckle tracking has been used with patient populations to evaluate contractility in areas affected by coronary artery stenosis or thrombosis and to evaluate recovery after reperfusion (PTCA: percutaneous transluminal coronary angioplasty). This method successfully identifies areas affected by reduced perfusion and confirms recovery of contractility in the affected areas after PTCA (Arbeille et al., 2012). Contractility in cardiac patients shows regional differences, dependent on the coronary arteries affected, requiring a 3D view of the ventricle for assessments. Astronauts, in contrast to PTCA patients, are unlikely to have coronary artery disease-related injuries to specific regions in the left ventricle. The expected changes are, rather, more likely to be global changes with spaceflight. In this way, the available 2D hardware on ISS was appropriate to use to assess LV contractility *in lieu* of using 3D hardware.

The objective of the present study was to evaluate left ventricle contractility before, during, and after 6 months spaceflight aboard the International Space Station (ISS). We hypothesize that the 2D speckle tracking would detect reductions in cardiac contractility during and after spaceflight, similar to the reductions previously observed with bedrest.

## Research design and methods

### Population

Ten astronauts (9 males, 1 female, age: 44 ± 3 years, height: 177 ± 5 cm, mass: 76 ± 11 kg) participated in the study. The protocol was approved by the University of Waterloo Office of Research Ethics (ORE #30362), Johnson Space Center Committee for the Protection of Human Subjects, NASA Human Research Medical Review Board, the European Space Agency Medical Review Board and Japanese Space Agency Research Ethics board (Study Protocol #Pro1222 for the Vascular Echo and Aging studies to which the present astronauts participated. NASA MPA116301606HR; FWA00019876) in accordance with the Declaration of Helsinki. Each participant was informed in detail about the experiment and gave informed consent before participating.

### Data collection

Echocardiography was conducted preflight (PRE), during the spaceflight on flight day 150 (FD150), and 4 days after the astronauts returned to Earth (R4). PRE and R4 assessments were conducted bedside by a trained sonographer with the astronaut resting supine while FD150 data were self-collected by the astronaut on the ISS *via* voice- and video-assisted remote guidance from a trained sonographer on the ground. For each assessment, the parasternal long axis and 4-chamber apical views were obtained for analysis.

For PRE and R4. data were collected on all astronauts using the ECHO device (CNES (Toulouse), Orcheo-LITE, Sonoscanner, Paris, France), using custom probes (Vermon, Tours) with custom driver software (Optimalog, Tours France). The device was equipped with a 3 MHz phased array (Arbeille et al., 2018). For the first four crew members recruited, FD150 inflight data were collected using the NASA HRF scanner (GE Logic Book, United States). The CNES Sonoscanner was used for the remainder of the astronauts as the Sonoscaner system was only installed on the ISS after the initial study recruitment.

### Data analysis

**FIGURE 1:**
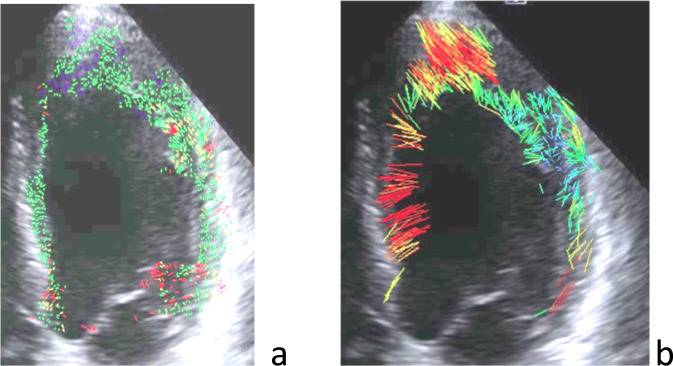
**(a)** Speckles distribution over the left ventricle myocardium at one moment during the cardiac cycle, and **(b)** the trajectory of each of these speckles during the entire cardiac cycle.

Custom software was developed to identify and track the speckles on the 2D ultrasound video (LIPADE laboratory, University of Paris Cité - France). This software was developed on an Ubuntu virtual machine (https://forum.ubuntu-fr.org), installed on a Windows PC computer. It was designed to detect the contours of the left ventricle and the speckles inside the left ventricle myocardium on a 2D echocardiographic video (Apical view). Each speckle detected was followed inside the myocardium for each frame of recorded video. Frame by frame speckle displacements were displayed and tracked over longer periods of time for the determination of displacement throughout the cardiac cycle (Figure 1). The software was specifically designed to assess recorded ultrasound video, not the raw radio frequency data, which allows for its use on 2D video recorded by any ultrasound device.

**FIGURE 2:**
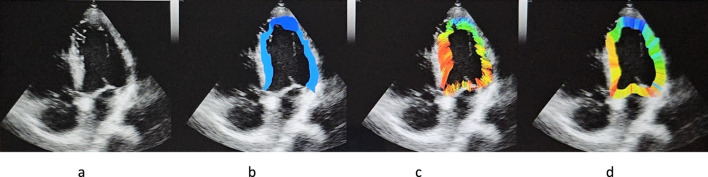
**(a)** Echocardiography of the left ventricle, **(b)** determination of the left ventricle contour, **(c)** displacement of the speckles, **(d)** projection of displacement along the radius launched from the left ventricle gravity center.

**FIGURE 3:**
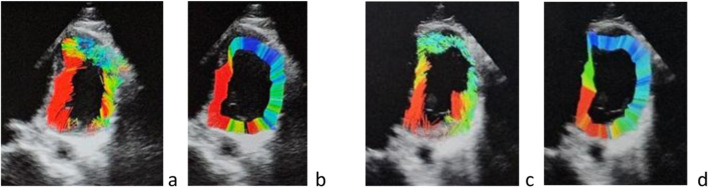
Example of reduced speckle displacement change postflight **(c)** compared to preflight **(a)**: **(a)** Speckle displacement **(b)** radial projection of these displacements on the radius generated from the center of the ventricle preflight, and **(c,d)** postflight. The red area preflight **(a,b)** is larger than on R4 postflight **(c,d)**. The area of high speed (long speckle trajectory) was less important on Figure 2C (postflight day +4) compared to Figure 2A (preflight). Same visual impression with the projection of the speckle trajectory on the radius generated from the left ventricle center. Less red and more blue area on Figure 2D compared to **(b)**.

For the current study, video of the left ventricle apical view was cut into a series of JPEG images (10–12 images) for one cardiac cycle using VirtualDub software. This sequence of images was then imported into the speckle tracking software. The software first displayed an image of the left ventricle in diastole on which a sonographer was required to mark (with a caliper) three points along the internal contour of the left ventricle myocardium on each side of the mitral valve and at the apex. These three points allow for the determination of the center of gravity of the ventricle. The software uses this point for the determination of the ventricle internal and external contours and the detection of the speckles between these 2 limits along 720 rays (Figure 2b). Then, on the same screen, the software displays the ultrasound image, the contour of the left ventricle, the speckle detected as colored points (Figure 1a), and the speckle displacement (trajectory) as colored segments (Figures 1b, 2c). Tracking was achieved based on Lucas-Kanade method, with displacements displayed as small line segments with color to represent the measured length of the displacement (for example, 1px = 0.48 mm and red color indicates the largest displacements) (Figure 2a). Another view is built (Figure 3d) that illustrates the contractility along each of the 720 radius lines generated from the center of the left ventricle. For every radius vector, if detected speckles have gone through the radius segment limited by the left ventricle contour, the radius line is colored according to the minimum displacement of these speckles with red representing the speckles that traveled the longest distance from the center.

In addition to the visuals displays described above, the software additionally provided the four following measurements: (1) the number of speckles identified on each frame of the sequence, (2) the number of speckles that were tracked between frames, and (3) the pixel displacements of the detected and tracked speckles. (4) The change in proportion of high displacement speckles (color display) was used to determine the area with the highest displacements (Figure 2). In this paper we report the change of these parameters between preflight, inflight and postflight (Figures 1–3).

Using the M-mode parasternal chamber view of the LV long axis the following parameters were measured on the time motion trace by 2 sonographers: stroke volume (SV, mL), ejection fraction (EF, %), left ventricular end diastolic volume (LVDV, mL), and myocardium thickness (Myoc, mm). The limits of the cardiac cavity on the Time motion trace were located manually by 2 sonographers and the image to be process randomized like in previous studies (Dorfman et al., 2007).

### Statistical analysis

The effects of spaceflight and recovery were assessed using a one-way repeated measure analysis of variance (SigmaPlot 12.5, Systat Software Inc., San Jose, CA). All variables passed tests for normality (Shapiro-Wilk test) and equal variance (Levene Median test). Significant main effects of spaceflight were further assessed using Tukey *post hoc* testing to evaluate pairwise comparisons. For all tests, significance was set at p < 0.05 with data reported as mean ± standard deviation.

## Results

**FIGURE 4:**
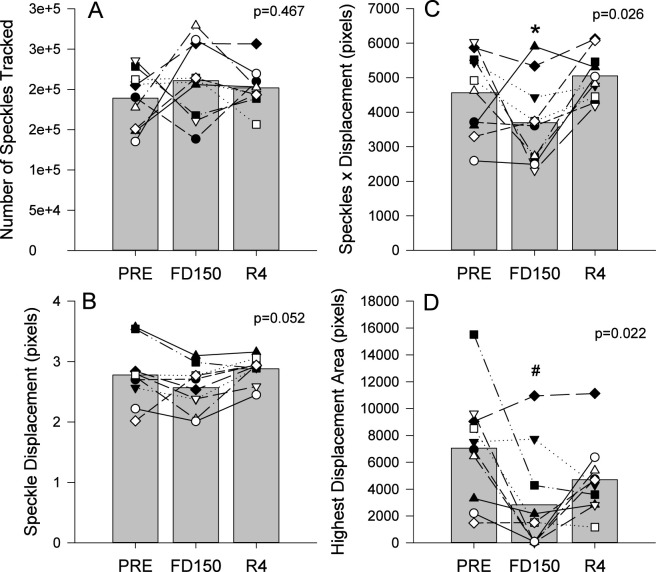
Mean (bars) and individual (symbols) measures of the number of tracked speckles **(A)**, the mean speckle displacement **(B)**, the product of tracked speckles and mean displacement **(C)** and areas of greater displacement **(D)**. Data are shown for preflight (PRE; n = 11), flight day 150 (FD150; n = 10), and 4 days after returning to Earth (R4; n = 11). Main effects of measurement time are indicated on the graphs. The * indicates a value different from R4 (p < 0.05) and # indicates a value different from PRE (p < 0.05). Lines connecting symbols are included to identify individual trends and do not represent changes in the variables over time.

Video sequences of the left ventricle apical view were successfully recorded preflight, inflight day 150), and postflight for all ten astronauts. The results of the speckle tracking processing showed that of the speckles identified, approximately 97% of those were followed by the software during the cardiac cycle. The number of tracked speckles was not different from preflight (Figure 4A; p = 0.467).

There was a trend towards reduced mean displacement with spaceflight (Figure 4B; p = 0.052) with 7 of the 10 astronauts appearing to show a reduction in displacement on FD150 and values returning to PRE levels on R4. The product of displacement by number of speckles (Figure 4C) and the size of the highest displacement area (Figure 4D) were both affected by spaceflight (p = 0.026 and p = 0.022 respectively). Post hoc analysis did not find a difference between PRE and FD150 displacement by speckle values (p = 0.168) but values appeared to be decreased in 7 of the 10 astronauts. Displacement by number of speckles values were lower on FD150 than R4 (p = 0.021) with no differences found between PRE and R4 (p = 0.542). In contrast, the highest displacement area was significantly reduced on FD150 from PRE (p = 0.017) but the area on R4 was not different from PRE (p = 0.227) or FD150Displacement by number of speckles values were lowe (p = 0.380).

**FIGURE 5:**
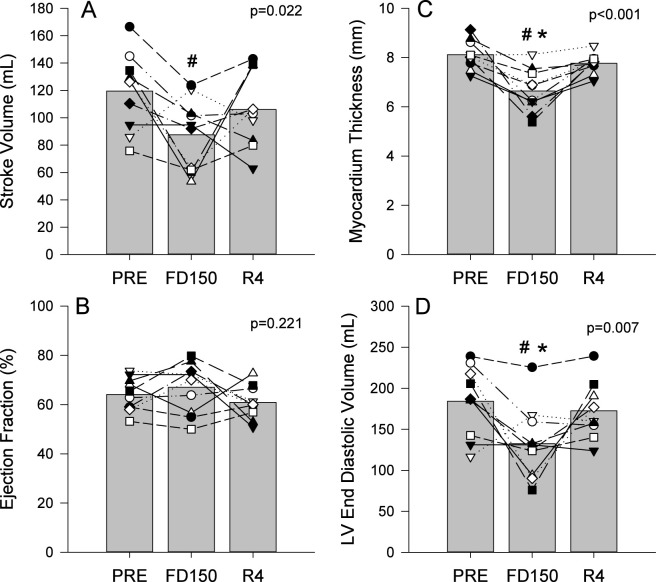
Mean (bars) and individual (symbols) measures of stroke volume **(A)**, ejection fraction **(B)**, myocardium thickness **(C)**, and left ventricle end diastolic volume **(D)**. Data are shown for preflight (PRE), flight day 150 (FD150), and 4 days after returning to Earth (R4). Main effects of measurement time are indicated on the graphs. The * indicates a value different from R4 (p < 0.05) and # indicates a value different from PRE (p < 0.05). Lines connecting symbols are included to identify individual trends and do not represent changes in the variables over time.

Stroke volume was found to be affected by spaceflight (Figure 5A; p = 0.022) with values lower on FD150 compared to PRE (p = 0.018) and no differences found between FD150 and R4 (p = 0.208) and PRE and R4 (p = 0.418). No effects of spaceflight were found for ejection fraction (Figure 5B; p = 0.221), but significant effects of spaceflight were found for both left ventricular end diastolic volume (Figure 5D; p = 0.007), and myocardium thickness (Figure 5C; p < 0.001). Myocardium thickness was reduced on FD150 compared to PRE (p < 0.001) and R4 (p < 0.001) with no difference between PRE and R4 (p = 0.369). Left ventricular end diastolic volume was lower on FD150 than both PRE and R4 (p = 0.007 and p = 0.039 respectively) and not different between PRE and R4 (p = 0.715).

## Discussion

The current study utilized speckle tracking to evaluate cardiac contractility with long duration spaceflight and recovery. In clinical practice, 4D ultrasound speckle tracking is used to evaluate regional differences in cardiac contractility due to reduced perfusion (Arbeille et al., 2012; Arbeille et al., 2014). However, as 4D sonography was unavailable on the ISS and microgravity exposure was expected to result in global changes to cardiac structure and function the present software was used to conduct speckle tracking on 2D ultrasound video. After 150 days in space, there was a trend towards reduced contractility (reduced speckle displacement, product of displacement and the number of speckles) and a significant reduction in the area with the greatest speckle displacement with a reduction in myocardium thickness and left ventricular end diastolic volume. However, individual differences in responses were evident.

To evaluate cardiac contractility with long duration spaceflight, three speckle tracking measures were utilized. Mean displacement of the speckles as a quantitative indication of the quality of left ventricle contractility, the product of mean displacement and the number of speckles tracked as an indication of the power of the whole contractility, and the portion of the myocardium with the greatest speckle displacements, as determined by the sonographer. While not statistically significant, potentially due to individual variability, the mean displacement of the speckles tracked showed a strong trend towards being reduced on FD150 and recovered on R4. Additionally, seven of the ten astronauts appeared to show reductions in contractility on FD150 and all but one appeared to show an increase on R4 compared to FD150. This trend is supported by both the quantitative assessment of the product of displacement and number of speckles tracked and the qualitative evaluation of the area with the greatest speckle displacement with both assessments indicating reduced contractility with spaceflight that is recovered 4 days after returning to Earth.

Previous spaceflight analogue studies have indicated cardiac atrophy with bedrest (Caiani et al., 2014; Dorfman et al., 2007). With spaceflight, analysis of the ultrasound radiofrequency signal has suggested morphological changes in liver tissue and the carotid artery wall which may be indicative of cellular remodeling (Arbeille et al., 2024a). In the current study, myocardium thickness was also reduced with spaceflight. However, in contrast to the bedrest studies, myocardium thickness recovered, for many astronauts, 4 days after returning to Earth. Similar pre-to post-flight observations with MRI data showed no changes in left ventricular mass after long duration spaceflight (Shibata et al., 2023). These observations would suggest that the observed reduction in cardiac contractility and myocardium thickness with spaceflight may be related to factors other than cellular remodeling.

Two factors may be contributing to the observed cardiac changes with spaceflight that recover quickly upon returning to Earth. First, while astronauts perform regular physical activity during spaceflight, physical activity levels are reduced compared to normal ambulation on Earth (Fraser et al., 2012) and may contribute to the observed cardiac responses (Nepomniashchikh et al., 1985; Shibata et al., 2023; Westby et al., 2016). Secondly, the effects of fluid shifts during spaceflight should also be considered as both spaceflight (Arbeille et al., 2021a; Arbeille et al., 2021b) and bedrest (Arbeille et al., 2024b) studies have shown increased jugular vein volume indicating a headward shift of fluid. However, the effects of fluid shifts with bedrest seem to be smaller as jugular vein volume is not increased to the same extent. Therefore, it would suggest that observed reductions in contractility are more related to reduced physical activity rather than fluid shifts.

Although overall physical activity may be reduced during spaceflight, exercise performed by the astronauts may account for some of the observed individual variation in responses. Previous work has shown that daily exercise prevented decreases in left ventricle volume, stroke volume, and ejection fraction after 60 days bedrest (Arbeille et al., 2008). Additionally, the exercise countermeasures prevented reductions in cardiac contractility observed with 21 days bedrest (Greaves et al., 2019). Therefore, it is possible that exercise performed by astronauts may have limited the impact of spaceflight on the contractility on FD150 and improved recover upon return to Earth.

Results from the current study also indicate a disconnect between cardiac contractility, stroke volume and ejection fraction during spaceflight. While left ventricular end diastolic volume was reduced with spaceflight, there was a small, but significant increase in ejection fraction that helped to maintain stroke volume despite the observation of reduced contractility. This is consistent with observations from coronary patients who did not increase ejection fraction (post PTCA) despite a 59% increase in contractility (Arbeille et al., 2012). This would suggest that additional variables need to be considered when interpreting the physiological effects of cardiac contractility changes determined with speckle tracking.

## Conclusion

The current study utilized novel speckle tracking software to evaluate cardiac contractility on astronauts using 2D ultrasound video. Combined, the three assessments of contractility suggest a mild reduction in contractility that is recovered 4 days after returning to Earth. The rapid recovery suggests that observed changes with spaceflight could be related to changes in physical activity and fluid shifts with microgravity and not changes in cellular remodeling.

## Data Availability

The datasets presented in this article are not readily available because medical privacy. Requests to access the datasets should be directed to the corresponding author.
